# The Effect and Mechanism of Regular Exercise on Improving Insulin Impedance: Based on the Perspective of Cellular and Molecular Levels

**DOI:** 10.3390/ijms26094199

**Published:** 2025-04-28

**Authors:** Tingran Zhang, Yongsen Liu, Yi Yang, Jiong Luo, Chen Hao

**Affiliations:** 1 College of Physical Education, Southwest University, Chongqing 400715, China; sharemix@swu.edu.cn (T.Z.); 19942339011@163.com (Y.L.); yangyi992416@163.com (Y.Y.); luojiong@swu.edu.cn (J.L.); 2 College of Physical Education, Chongqing University, Chongqing 401331, China

**Keywords:** hyperglycemia, insulin resistance, information transfer pathway, regular exercise, molecular biology

## Abstract

Insulin resistance is more common in the elderly, and with the improvement in people’s living standards and changes in lifestyle habits, the incidence of insulin resistance in other age groups is also increasing year by year. Overweight and obesity caused by abnormal fat metabolism or accumulation can significantly reduce glucose intake, which is the direct cause of insulin resistance and the trigger for the occurrence and development of type II diabetes. This article reviews and analyzes relevant literature on empirical research on the effect of regular exercise on improving insulin resistance. It was found that the most important step in carbohydrate metabolism is the translocation of glucose transporter 4 (GLUT4) to the cell membrane, carrying water-soluble glucose through the lipid soluble cell membrane to complete carbohydrate transport. The process of glucose transporter protein translocation to the cell membrane can be driven by two different signaling pathways: one is the insulin information transfer pathway (ITP), the second is to induce the ITP of monophosphate-activated protein kinase (AMPK) through hypoxia or muscle contraction. For type II diabetes patients, the insulin signal transmission pathway through insulin receptors (IRS_1_, IRS_2_) and phosphatidylinositol 3-kinase (PI3K) (PI3K) is damaged, which results in the decrease in glucose absorption stimulated by insulin in skeletal muscle, while the noninsulin signal transmission pathway of AMPK in these patients is normal. It can be seen that regular exercise can regulate glucose intake and the metabolism of skeletal muscle, improve insulin resistance, reduce fasting blood glucose and glycosylated hemoglobin in diabetes patients, and thus, effectively regulate blood glucose. However, many steps in the molecular mechanism of how exercise training improves systemic insulin resistance are still not fully understood, and further discussion is needed in the future.

## 1. Introduction

Modern scientific and technological civilization has changed people’s way of life, resulting in a significant decline in people’s sports. The popularity of the sedentary lifestyle, and high-calorie and high-fat diet has changed the dietary habits of people, and further promoted chronic diseases such as cerebrovascular disease, heart disease, hypertension, liver disease, and diabetes, which have become the mainstream of the top ten causes of death in China in recent years [1,2,3]. According to the latest survey, the current incidence rate of diabetes in China is about 5%, which means about 70 million people. According to the prediction of the World Health Organization, by 2025, the number of diabetes patients in the world will reach 300 million, of which 230 million will be in developing countries [4,5]. The International Diabetes Federation (IDF) released the world’s eighth edition of the diabetes map, which shows that the death toll from diabetes in China in 2017 exceeded the first killer of cancer. The risk factors of cardiovascular diseases, hypertension, diabetes, and obesity are among the top five high-risk factors, and these factors are mutually causal and closely related, which has become an important reason for the high incidence of cardiovascular events in China in recent years [6,7,8].

Insulin resistance (IR) is the central link in the occurrence and development of cardiovascular and cerebrovascular diseases, hyperlipidemia, diabetes, fatty liver, and other diseases. A high-fat diet (HFD) and sedentary lifestyle are important risk factors for IR. Numerous studies have shown that HFD-induced insulin resistance (IR) may be associated with ectopic deposition of skeletal muscle fat [9], and the relationship between sedentary behavior and IR is mainly mediated by skeletal muscle. Therefore, exercise training can effectively prevent and improve these diseases [10]. Its main mechanism is to affect the secretion of skeletal muscle adipokines, activate insulin-dependent glucose transport, improve mitochondrial aerobic metabolism, and increase their biogenesis [11,12]. Insulin resistance is a gradually developing process that can usually be divided into four stages [13]. Phase 1: Early insulin resistance. At this stage, the skeletal muscle’s response to insulin begins to decrease, but blood sugar levels remain within the normal range, whether on an empty stomach or after a meal. This is because pancreatic beta cells compensate by secreting more insulin to maintain blood sugar balance. Phase 2: Pre-sugar stage. This stage is a critical period for reversing insulin resistance. Insulin resistance further develops and glucose tolerance begins to be impaired, indicating that the function of pancreatic beta cells is beginning to malfunction. The main manifestation is that fasting or postprandial blood glucose exceeds the normal range, insulin resistance and pancreatic beta cell dysfunction coexist, fasting blood glucose regulation is abnormal between 5.6 and7.0 mmol/L, or postprandial two-hour blood glucose is between 7.8 and 11.1 mmol/L. The third stage: Type II diabetes. At this stage, insulin resistance further worsens, pancreatic function is impaired, with a fasting blood glucose > 7.0 mmol/L or postprandial two-hour blood glucose > 11.1 mmol/L. According to the new international standard in 2024, a one-hour postprandial blood glucose ≥ 8.6 mmol/L is defined as pre-glucose, and a fasting blood glucose ≥ 10.6 mmol/L is defined as type II diabetes. The fourth stage: Late diabetes. At this stage, pancreatic beta cell function fails and the ability to secrete insulin is completely insufficient, relying only on exogenous insulin to control blood sugar. In clinical practice, type II diabetes can be divided into insulin resistance type and insulin secretion insufficiency type according to the patient’s condition. Insulin resistance is characterized by insulin resistance and insufficient insulin secretion, and patients may have symptoms such as obesity and pre-meal hypoglycemia. The insulin secretion deficiency type is characterized by mild insulin resistance and symptoms such as frequent urination and thirst. Therefore, the treatment strategy for diabetes is no longer just to deal with late complications of diabetes but to prevent the further deterioration of insulin resistance in advance [14,15]. It can be seen that diabetes patients are unable to perform normal functions due to insufficient endocrine of the hormone insulin carrier necessary for carbohydrate metabolism, abnormal secretion time, and the reduction in insulin receptors on the cell surface of the main organs of insulin action such as the liver, muscle, and adipose tissue.

From a physiological perspective, aerobic exercise can improve skeletal muscle blood flow and mitochondrial biogenesis, and enhance the stability of key protein translation in the insulin signaling pathway, including increasing GLUT4 content, enhancing glycogen synthase and hexokinase activity, etc. [16]. Resistance exercise can promote muscle hypertrophy and, overall, enhance the body’s glucose metabolism ability [17]. In addition, studies have shown that a single session of resistance training can enhance insulin sensitivity for up to 24 h [18,19]. This effect may be partly due to a decrease in the storage of triglycerides (IMTG) in skeletal muscle, while long-term aerobic exercise training may increase IMTG levels [20], suggesting that different training methods have different effects on skeletal muscle fat content and metabolism. In addition, the effects of single training and multiple training may differ, and numerous studies have shown that single endurance training can also reduce IMTG content [21]. Therefore, improving insulin resistance through exercise is not only related to its impact on skeletal muscle fat content, but more importantly, it may enhance the ability to metabolize fatty acids.

However, on the other hand, it was found that most diabetes patients lack long-term exercise. According to the recommendation of the U.S. Department of Health on the amount of exercise required, only about 22% of adults can maintain a healthy level of physical activity, while less than 10% of adults can ensure sufficient exercise intensity and amount to improve their cardiopulmonary fitness [22]. Statistics at home and abroad have significantly pointed out that the amount of physical activity of modern people is obviously insufficient, and the amount of physical activity is closely related to the occurrence of chronic diseases, including heart disease, some cancers, diabetes, osteoporosis, stroke, obesity, back pain, depression, and other emotional disorders. A large number of studies have shown that [23,24,25] exercise is one of the three major elements for the prevention and treatment of diabetes. Regular exercise can effectively improve the blood lipids of patients with type 2 diabetes, reduce coagulation, enhance cardiopulmonary endurance, improve the ability of cells to receive insulin, eliminate insulin resistance, reduce blood glucose, and achieve the effect of preventing and controlling diabetes. Since the vast majority of diabetes patients are also obese, have coronary heart disease, cerebrovascular disease, or are hypertension patients, it seems that heredity is not the main cause of type 2 diabetes. The main cause of type 2 diabetes is the decrease in insulin sensitivity caused by the increase in fat distribution, blood lipid value, and exercise ability [26,27]. If the insulin signaling pathway of patients with diabetes or insulin resistance is damaged, the effect of exercise training will be greatly affected. However, the noninsulin signaling pathway of these patients is still intact, and the glucose transport capacity of muscle tissue increases after exercise training. This paper systematically reviews the empirical study of regular exercise on improving insulin-stimulated carbohydrate absorption, and discusses the mechanism by which exercise training may induce the AMPK signaling pathway to increase carbohydrate transport, to provide an important reference for how to prevent and treat insulin resistance and related metabolic syndrome in the clinic.

## 2. Possible Mechanisms of Insulin Resistance

Because of their water solubility, sugars cannot pass through the lipid bilayer structure of the cell membrane by themselves. They need to be carried and transported with the help of special proteins on the surface of cell membranes before they can enter the cell for oxidative decomposition and energy supply. These glucose-transporting proteins are called the GLUT family and are named according to the order in which they are found. Among them, glucose transporter 4 (GLUT4) protein is the most important protein in skeletal muscle [8]. The research on the pathogenesis of diabetes has shifted from insulin receptor damage to the insulin receptor signaling path [4,28,29].

### 2.1. Molecular Mechanisms Regulating Glucose Absorption

**Figure 1:**
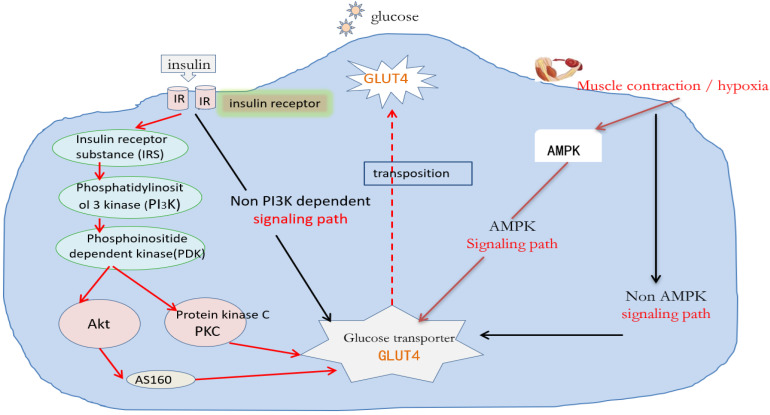
Molecular mechanisms regulating glucose absorption. Notes: The dashed line represents an uncertain path, the solid line represents a determined path, and arrows of different colors represent different action paths.

Transmembrane tyrosine kinase is an insulin receptor located on the cell membrane. Its molecular structure outside the cell membrane will cause phosphorylation of the molecular structure inside the cell membrane after binding with insulin, and then attract insulin receptor substrate (IRS). The IRS molecule mainly acts as a parking protein and can adsorb other proteins with SH2 molecular fragments, The most important thing is to attract phosphatidylinositol 3-kinase (PI3K). PI3K is a very important kinase, which can catalyze the formation of phosphatidylinositol 3-phosphate and regulate phosphoinositide-dependent kinase (PDK). PDK can activate several protein kinases of downstream serine and threonine kinases, such as prototype protein kinases A, B, C, and G (i.e., PKA, PKB, PKC, and PKG) [30,31,32]. Akt (also known as PKB) is a very important signaling pathway molecule for maintaining survival in cells. It can activate the downstream AS160 molecule. It is a small G protein that can assist GLUT4 in the cytoplasm to form small vesicles, transport and translocate to the cell membrane, and perform tasks [33]. The path of insulin binding with insulin receptors via PI3K is a very important signaling path for insulin. Recently, it has been reported that some insulin information can be transmitted without PI3K, but the transmission molecules of this part are still unclear [34,35] (see the black arrow path mechanism on the left of Figure 1).

AMP-activated protein kinase (AMPK) is an important substance that undertakes the noninsulin signaling pathway [13,14,36]. Its activation is affected by the ratio of adenosine monophosphate (AMP) and adenosine triphosphate (ATP) in cells. When cells contract under hypoxia, they must consume intracellular ATP and ADP and produce more AMP, which will induce the activity of AMPK, especially since the heterogeneous body of AMPK α_2_ is more affected by AMP concentration [14,37,38]. The activated AMPK can promote the translocation of GLUT4 to the cell membrane to complete the corresponding tasks, but its molecular mechanism is still unclear. The AMPK pathway can drive GLUT4 to absorb glucose without insulin, so it is called the noninsulin signaling pathway. The latest research shows that after blocking the AMPK pathway, muscle contraction can still increase glucose absorption, which indicates that exercise or muscle contraction can induce transfer pathway other than AMPK to increase glucose absorption [22,39]. Further in-depth study found that this transfer pathway other than AMPK mainly depends on mitogen-activated protein kinase (MAPK). It is a group of serine–threonine protein kinases that can be activated by different extracellular stimuli, such as cytokines, neurotransmitters, hormones, cell stress, and cell adhesion. It is an important transmitter for signal transmission from the cell surface to the nucleus [23,40]. MAPK, a molecular transfer pathway, is related to gene transcription in the nucleus [24,25,41], which can increase the production of many key proteins, including GLUT4. It can be seen that the insulin signaling mechanism of patients with type 2 diabetes is damaged; that is, the expression of messenger ribonucleic acid (mRNA) or protein of many molecules in the signaling path is damaged, but the noninsulin signaling mechanism of AMPK in these patients is not damaged. Therefore, how to activate the AMPK signaling path is the key entry point for drug and exercise therapy of patients with insulin resistance and diabetes.

Metformin is a drug commonly used in the treatment of type II diabetes. It has the effect of regulating blood sugar levels and has a certain impact on muscles. It is mainly produced through four mechanisms for metabolic processes in the body, which have potential effects on muscle health. (1) Metformin mainly improves insulin sensitivity by activating AMP-activated protein kinase (AMPK), which in turn promotes glucose uptake by muscle cells and helps provide more energy for muscle use. (2) Metformin can increase the oxidation of fatty acids and reduce liver fat production, which is beneficial for promoting fat oxidation and decomposition, reducing fat accumulation in muscles. (3) Metformin may protect muscle proteins by reducing specific protein degradation pathways, thereby maintaining or increasing muscle mass. (4) Metformin can regulate immune system activity and alleviate chronic inflammatory reactions in muscles, thereby protecting muscle cells from damage. However, when using metformin, it is necessary to combine individual circumstances and medical guidance to avoid the occurrence of side effects.

### 2.2. Molecular Mechanism of Insulin Resistance Induced by Lipid Accumulation in Muscle Tissue

**Figure 2:**
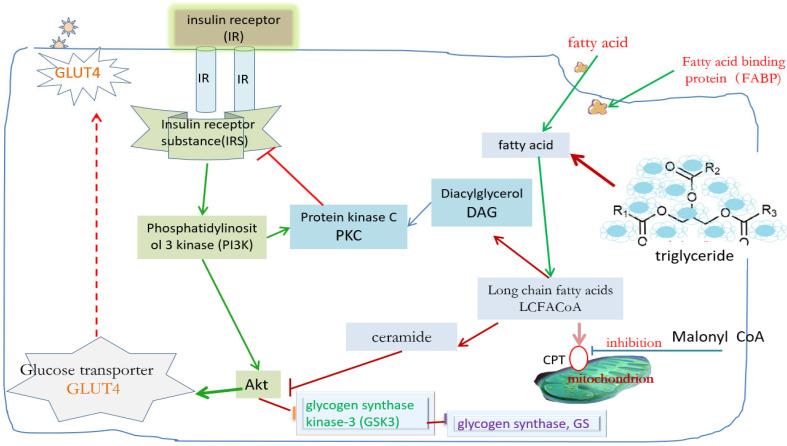
The corresponding mechanism of lipid accumulation and possible insulin resistance. Notes: The dashed line represents an uncertain path, the solid line represents a determined path, and arrows of different colors represent different action paths.

It is not difficult to find from Figure 2 that in addition to diffusing through the cell membrane into the cell, extracellular fatty acids (FA) can also be brought into the cell by binding with the fatty acid binding protein (FABP) family in the cytoplasm [31,32,33,34]. These FABPs with specific functions play a role similar to GLUT4. After entering the cell, fatty acids are mainly converted into long-chain fatty acids, some of which are converted into triglycerides and stored in the cell, others are broken down into smaller molecules, such as diacylglycerol (DAG) or acyl sphingosine, and others enter the mitochondria for oxidative decomposition for energy supply. At present, there are 14 kinds of FABP reported. In addition to carrying fatty acids to specific targets, they are also involved in regulating intracellular fat metabolism and gene expression. For example, FABP_2_ in the small intestine can help regulate the absorption of fatty acids and the secretion of chyle droplets after diet [35,42]; FABP_3_ in the heart is a necessary protein for maintaining normal myocardial fatty acid metabolism [43], which is also related to fatty acid uptake in muscle tissue [44]; FABP of the liver can transmit information into the nucleus after binding with fatty acids and act with peroxisome proliferator-activated receptor (PPAR) in hepatocytes [45,46]; and FABP_4_ (also known as ap_2_) in adipose tissue and immune cells can affect insulin sensitivity, fat metabolism, and lipolysis [47].

The amount of energy stored in adipose tissue can affect the sensitivity of muscle tissue and the liver to insulin [48,49]. When the body stores too many triglycerides, the expanded fat cells will automatically release some inhibitory feedback information to inform the body to reduce energy absorption [50,51]. Adipocytes play an important role in the monitoring of human energy storage because leptin secreted by adipocytes is transmitted through the blood to the hypothalamus of the brain, which combines with the receptor there to make the brain feel full, to reduce dietary intake and reduce weight. In addition to leptin, adipose tissue also secretes several important hormones [49,50], including acylation stimulating protein (ASP), adiponectin, plasminogen activator inhibitor-1 (PAI-1), lipase, resistin and tumor necrosis factor (TNF-α), cytokine 1 (IL-1), cytokine 6 (IL-6), etc. However, when too many fatty acids enter cells, molecules such as long-chain fatty acids, diacylglycerol, and acyl sphingosine will inhibit the role of some kinases in the normal insulin signaling pathway [52]. For example, diacylglycerol can activate PKC and interfere with the tyrosine phosphorylation of IRS, transforming it into serine phosphorylation, thus affecting the normal insulin signaling path. Acyl sphingosine interferes with the inhibitory effect of Akt on glycogen synthase kinase-3 (GSK3) and affects the effect of glycogen synthase (GS). In addition, scholars found that the ability of lipid oxidation in muscle tissue of obese or insulin-resistant patients decreased significantly, which significantly reduced the fatty acids metabolized into mitochondria. Therefore, the whole phenomenon becomes that there is too much triglyceride accumulation in the cytoplasm, which affects the normal insulin signaling path, resulting in GLUT4 being unable to receive the correct information, translocate to the cell membrane, carry glucose molecules into the cell, and finally increase blood glucose [14,31]. It can be seen that excessive lipid accumulation in muscle tissue and the liver will lead to metabolic changes, including nutrient competition, changes in leptin regulation, changes in intracellular signaling molecules or gene transcription, and finally diabetes.

### 2.3. Oxidative Stress and Inflammatory Mechanism Related to Insulin Resistance

When the cell is in a condition of high glucose or excessive free fatty acids, it will drive the mitochondria to release peroxides (ROS). ROS not only directly causes damage to the protein, fat, and deoxyribonucleic acid (DNA) in the cell but also causes late complications of diabetes [53,54], and ROS itself can be used as a signaling molecule to start several signaling paths [55], including the NF-κB pathway, p38 MAPK pathway, JNK-SAPK pathway, etc. These signaling pathways are related to intracellular apoptosis or the inflammatory response. These signaling pathways can drive several different products to cause cell inflammation or proliferation, which may cause insulin resistance or damage to the pancreas β cells [55]. In addition, these signaling paths can trigger downstream serine to inhibit the tyrosine phosphating reaction of IRS [56], indirectly hinder the normal insulin signaling path, resulting in the inability of GLUT4 to translocate to the cell membrane to perform work, and then lead to intracellular insulin resistance.

Clinical studies have found that when the body is invaded by foreign objects, immune cells will secrete cytokines to resist and cause the inflammatory reaction of the body, so as to change the metabolic state in cells and provide the energy source for immune cells to work, so that the cells are in a state of high glucose or high fat [57,58]. Therefore, when the body has been in the case of chronic inflammation, the metabolic environment in cells will always be in a state of high glucose or high fat. These states will increase the production of cell peroxides, cause oxidative pressure on cells, and then drive the activation of the previously mentioned signaling path, resulting in hyperlipidemia, insulin resistance, and other phenomena. More and more clinical evidence link inflammation, obesity, insulin resistance, and cardiovascular disease, and some inflammatory indicators such as C-reactive protein (CRP) are often used to predict whether diabetes patients have cardiovascular disease [15,59,60].

To sum up, the mechanism of inflammation and oxidative stress leading to insulin resistance is still unclear, but at least it can be speculated that cytokines may activate different signaling pathways and then affect gene transcription in the nucleus, or increase intracellular oxidative pressure to form downstream serine, inhibit the IRS tyrosine phosphating reaction, and indirectly hinder the normal insulin signaling pathway. This reuces the ability of GLUT4 translocation to the cell membrane, resulting in intracellular insulin resistance. This requires more experiments in the future to test these hypotheses.

## 3. Possible Mechanism of Exercise Training Improving Muscle Insulin Resistance

### 3.1. Effect of Exercise on the Insulin Signaling Pathway

After exercise training, the molecular activity upstream of the insulin signaling path changes significantly [31,61], which greatly improves the ability of the AMPK signaling path to translocate GLUT4. It has been found that IRS is a very important upstream referral molecule in the insulin signaling path. It has four kinds of heterogeneous bodies, including IRS_1_ and IRS_2_ in the human body. IRS_1_ is mainly related to signaling in muscle tissue, and IRS_2_ is related to β cell development and metabolism in the liver. This study found that the effect of exercise training on the insulin signaling path does not increase the gene transcription or protein production of IRS_1_ but improves the efficiency of each IRS_1_ molecule and increases its efficiency of transmitting information downward [26,49]. On the other hand, exercise training can significantly enhance the mRNA and protein expression of IRS_2_ and tyrosine phosphating ability [62] because PI3K activity increases significantly after exercise training, and PI3K can receive information from IRS_1_ or IRS_2_, to promote glucose absorption [62,63,64].

The increase in PI3K activity after exercise training is also related to the increase in glucose absorption after exercise. PI3K can receive messages from IRS_1_ or IRS_2_, and insulin action and IRS_1_ activation of PI3K occur simultaneously after exercise training [56,61]. IRS_1_ is the main molecule that transmits insulin messages to PI3K during exercise training. Therefore, the activation of PI3K by IRS_2_ also significantly increases after exercise. Due to the different effects of exercise training on the mRNA and protein expression of IRS_1_ and IRS_2_, scholars believe that the response of IRS_1_ and IRS_2_ to exercise training is specific, rather than a purely complementary compensatory relationship [54,55]. An animal experiment compared the changes in insulin signaling pathways between IRS_2_ knockout mice and wild-type mice after treadmill exercise [63]. The results showed that the increase in PI3K activity in IRS_2_ knockout mice after exercise was less than that in wild-type mice, indicating that the increase in PI3K activity during exercise training did come partially from IRS_2_, rather than being completely compensated by IRS_1_. However, further research is needed to explore the physiological significance of IRS_2_ signaling.

### 3.2. Effect of Exercise on AMPK

The effect of exercise training on improving the impaired insulin signaling pathway is limited, but the noninsulin signaling pathway of these diabetes patients is intact. Relevant studies have found that during exercise, a large amount of ATP decomposes and provides energy, which will produce a large amount of AMP, to activate AMPK. Through the AMPK path, GLUT4 can be translocated to the cell membrane and increase glucose transport [65,66,67]. AMPK plays a key role in the mechanism of improving glucose homeostasis through exercise training. It has two heterogeneities (α_1_ and α_2_), and low and medium-intensity aerobic exercise training can significantly increase AMPKα_2_, while AMPKα_1_ did not change [44,68]. The performance of both heterosomes increased under anaerobic exercise. In addition to being directly induced by exercise or muscle contraction, AMPK can simulate the translocation of GLUT4 to the surface of the cell membrane during exercise by stimulating the chemical information molecules of AMPK, to reduce blood glucose. At present, AMPK-stimulating drugs have been successfully developed, such as 5-amino-4-carboxamide riboside (AICAR), which can act on hepatocytes, adipocytes, and muscle tissues to cause similar muscle contraction or hypoxia to produce AMP and induce AMPK activity, to move GLUT4 to the surface of the cell membrane and transport glucose, and to reduce blood glucose and improve the expression of muscle GLUT4 protein. The effect is similar to that of insulin sensitivity induced by exercise training.

### 3.3. Effect of Exercise on MAPK

Previous studies found that the glucose absorption capacity of mice decreased only partially after exercise by knocking out the AMPK gene of mice, indicating the existence of a non-AMPK message transfer pathway. The MAPK pathway is responsible for many processes of cell proliferation and differentiation, and MAPK itself is a large molecular family, with at least three parallel signaling pathways [23,61], including extracellular regulated protein kinases, such as ERK1/2 or P42/44MAPK, and P38MAPK and c-Jun NH_2_ kinase. Research findings [31,39] suggest both acute and chronic exercise can directly activate these MAPK transfer pathways, thereby improving the performance of relevant molecules in these signaling pathways. However, the role of these signaling pathways in patients with insulin resistance or diabetes is still unclear. In addition, AMPK is involved in a variety of gene expressions related to exercise, including increasing GLUT4 mRNA and protein expression, increasing granuloglandular enzymes, and increasing hepatic glucose storage. It can also activate P38MAPK [29], which seems to suggest that there may be some interaction between AMPK and the MAPK pathway, and further research is needed in the future to reveal the relevant mechanism.

### 3.4. Effect of Exercise on Glucose Transporter

Studies have shown that improving the expression of GLUT4 in muscle can increase the sensitivity of the body to insulin [69]. Animal experiments have found that exercise training can increase the amount of overall GLUT4 protein by 1.7–2.3 times [70], and the increase in GLUT4 protein is related to the increase in GLUT4 mRNA transcription after exercise training, but the mRNA transcription will return to the reference point 24 h after exercise; however, the protein quality can continue to increase for several days [71,72]. There are two positions on the intron of the GLUT4 gene that can bind to myocyte-enhancing factor-2 (MEF2) and GLUT4-enhancing factor (GEF) [6,73]. These two proteins are important molecules that regulate the increase in GLUT4 gene transcription after exercise, and the change in the intracellular energy state and calcium concentration during exercise may be an important inducement to induce these promoting factors to bind to the GLUT4 gene. In conclusion, exercise training may not be able to change the pathway of GLUT4 translocation driven by insulin signaling pathway molecules, but it can affect GLUT4 translocation through the transfer pathway of AMPK. Therefore, it can be considered that the effect of exercise training on the overall GLUT4 translocation is partly compensated by increasing the yield of the GLUT4 protein.

### 3.5. Effects of Exercise on Hepatic Glucose Synthase and Insulin Sensitivity

Whether in animal experiments or human experiments, insulin transport increases during exercise, but the upstream molecules of the insulin signaling path do not increase [74], which may be because the consumption of liver sugar stored in muscle during exercise can enhance the role of liver glucose synthase, to promote the glucose outside the cell membrane to easily enter the cell, to improve glucose transport. However, the relevant molecular mechanism is not clear. Scholars speculate that animals with low hepatic glucose storage during exercise can accelerate the transfer pathway of AMPK, or increase the activation function of Akt in the insulin transfer pathway, thereby activating the function of hepatic glucose synthase [75,76]. Exercise training can enhance the induction of GLUT4 to AMPK, improve insulin signaling ability, increase nitric oxide (NO) synthesis, and increase calcium ion concentration [71]. During muscle contraction, AMPK is increased and activated due to the conversion of ATP into AMP. In addition, muscle contraction has been found to increase the concentration of NO and calcium ions. In addition to the insulin signaling pathway, AMPK, NO concentration, and calcium ions also directly stimulate the translocation of GLUT4 to the cell surface [69], assisting in the transport of blood glucose and reducing insulin impedance.

### 3.6. Effect of Exercise on Muscle Fatty Acid Absorption

Aerobic exercise can increase the oxidation capacity of muscle tissue. This effect often comes from the utilization rate of free fatty acids in the blood and the improvement rate of fatty acid binding proteins [77,78]. Studies have shown that the mRNA and protein of a fatty acid binding protein (FAT/CD36) are significantly increased after chronic aerobic exercise training, indicating that the utilization and metabolism of fatty acids in muscle are improved. Many oxidation-related leptin activities in muscles with insulin resistance become slower, while the volume of glandular granules in muscle cells becomes smaller [79]. In addition, exercise training significantly reduces fat accumulation in muscle tissue and speeds up the clearance of intracellular triglycerides, which also helps to reduce insulin resistance [37].

To sum up, the existing empirical research shows that long-term aerobic exercise and resistance exercise can bring positive benefits to the blood sugar regulation of diabetes patients [39,67,69]. In type I diabetes, aerobic training can increase cardiopulmonary compliance, reduce blood lipids (LDL) and triglyceride, and improve vascular endothelial proliferation [42,50]. In type II diabetes patients, aerobic training can reduce glycosylated hemoglobin (HbA1c), triglyceride, and insulin resistance [66]. Resistance exercise can increase muscle mass and reduce body fat, and has a positive impact on blood glucose regulation. Intermittent aerobic training is more effective in improving blood glucose control than continuous aerobic training, while high-intensity interval training (HIIT) reduces the total exercise volume but still has a positive impact on blood glucose control and insulin sensitivity. However, the optimal intensity and frequency of exercise are still uncertain. The American Diabetes Association recommends that moderate-intensity aerobic exercise should be carried out at least three times a week for at least 150 min in total according to the guidelines for improving diabetes through exercise. Walking, yoga, swimming or water aerobic exercise, leisure physical activities, and other forms of exercise are all suitable projects [72,74]. Therefore, selecting appropriate types of exercise based on individual needs, or considering scheduling two different types of exercises simultaneously in the training plan is important. However, at present, there are few pieces of literature on resistance combined with aerobic exercise in the pre-diabetes population, and more literature is needed to explore its benefits, and further compare the effects of aerobic exercise, resistance exercise, and their combination on blood glucose regulation. Finally, when arranging exercise training plans, attention should be paid to the intensity to avoid the negative impact of high-intensity exercise training on blood sugar regulation.

## 4. Conclusions

(1) Insulin resistance is the common root of type 2 diabetes and many chronic diseases. The noninsulin signaling path of AMPK in patients with insulin resistance or diabetes is still intact. Therefore, the research and development of drug therapy should focus on how to induce the AMPK path to drive the smooth translocation of GLUT4, to improve insulin sensitivity.

(2) Regular exercise can effectively prevent insulin resistance and increase the functions of several key proteins in the insulin signaling path and AMPK path. However, there are still many key nodes in the molecular mechanism of how exercise training can improve systemic insulin resistance that need to be further discussed in the future.

## Data Availability

Not applicable.
